# Estimated glomerular filtration rate and postoperative mortality in patients undergoing non-cardiac and non-neuron surgery: a single-center retrospective study

**DOI:** 10.1186/s12893-020-00958-7

**Published:** 2021-03-06

**Authors:** Xueying Luo, Sujing Zheng, Baoer Liu, Liping Yang, Ya Li, Feng Li, Rui Gao, Haofei Hu, Jinsong He

**Affiliations:** 1 Department of Plastic and Reconstructive, Shenzhen University, No. 3688 Nanhai Avenue, Nanshan District, Shenzhen, 518000 Guangdong China; 2 Department of Thyroid and Breast Surgery, Shenzhen Second People’s Hospital, No. 3002, Sungang West Road, Futian District, Shenzhen, 518000 Guangdong China; 3 Department of Breast Thyroid Surgery, Shenzhen University, No. 3688 Nanhai Avenue, Nanshan District, Shenzhen, 518000 Guangdong China; 4 Department of General Medicine, Shenzhen University, No. 3002, Sungang West Road, Futian District, Shenzhen, 518000 Guangdong China; 5 Department of Nephrology, Shenzhen Second People’s Hospital, No. 3002, Sungang West Road, Futian District, Shenzhen, 518000 Guangdong China; 6 Department of Breast Thyroid Surgery, Shenzhen Breast Cancer Research and Treatment Research Center, Peking University Shenzhen Hospital, 1120 Lianhua Road, Futian District, Shenzhen, 518000 Guangdong China

**Keywords:** Estimated glomerular filtration rate, Postoperative 30-day mortality, Surgery, U-shaped curve

## Abstract

**Background:**

There is limited evidence to clarify the specific relationship between preoperative estimated glomerular filtration rate (preop-eGFR) and postoperative 30-day mortality in Asian patients undergoing non-cardiac and non-neuron surgery. We aimed to investigate details of this relationship.

**Methods:**

We reanalyzed a retrospective analysis of the clinical records of 90,785 surgical patients at the Singapore General Hospital from January 1, 2012 to October 31, 2016. The main outcome was postoperative 30-day mortality.

**Results:**

The average age of these recruited patients was 53.96 ± 16.88 years, of which approximately 51.64% were female. The mean of preop-eGFR distribution was 84.45 ± 38.56 mL/min/1.73 m^2^. Multivariate logistic regression analysis indicated that preop-eGFR was independently associated with 30-day mortality (adjusted odds ratio: 0.992; 95% confidence interval [CI] 0.990–0.995; P < 0.001). A U-shaped relationship was detected between preop-eGFR and 30-day mortality with an inflection point of 98.688 (P for log likelihood ratio test < 0.001). The effect sizes and confidence intervals on the right and left sides of the inflection point were 1.013 (1.007 to 1.019) [P < 0.0001] and 0.984 (0.981 to 0.987) [P < 0.0001], respectively. Preoperative comorbidities such as congestive heart failure (CHF), type 1 diabetes**,** ischemic heart disease (IHD), and anemia were associated with the odds ratio of preop-eGFR to 30-day mortality (interaction P < 0.05).

**Discussion:**

The relationship between preop-eGFR and 30-day mortality is U-shaped. The recommended preop-eGFR at which the rate of the 30-day mortality was lowest was 98.688 mL/min/1.73 m^2^.

## Background

The unmet global burden of surgical disease is enormous [1] With the popularity of surgery, it is especially important to optimize the safety of the operation [2]. 30-day mortality is one of the most important indicators of perioperative mortality (POMR) and can be used to effectively indicate the safety of surgery and the risk of postoperative complications [3]. Preoperative renal dysfunction is a ac-knowledgeable risk factor for postoperative mortality, and the risk of patients with moderate to severe kidney insufficiency increases dramatically [2, 4]. Estimated glomerular filtration rate (eGFR), describing filtrate flow through the kidneys, is a universal surrogate indicator for assessing renal function. It has been widely used in the clinical diagnosis of chronic kidney disease (CKD) [5].

Studies have shown that preop-eGFR is a moderately effective predictor of 30-day mortality in hospitalized surgical patients [4]. However, the current research population is mainly concentrated on patients who have undergone critical surgery such as cardiac surgery [6–14], lack of other surgeries. The ethnicity of these studies is also rarely related to Asians. Moreover, the current studies mainly focus on the relationship between preop-eGFR and perioperative mortality in patients with renal insufficiency [7, 15], but several reports address high preop-eGFR levels or any other asymptomatic patient. Only Takashi Ui at.al made it clear among patients undergoing gastrointestinal malignancies that high preop-eGFR is associated with poor surgical outcomes, and also indicated a U-shaped relationship of preop-eGFR and 30-day mortality [16]. However, data related to non-cardiac and non-neuron surgery are scarce.

Our study was designed to explore the details of relationship between preop-eGFR and 30-day mortality in Asian patients undergoing non-cardiac and non-neuron surgery. Not only limited to patients with renal insufficiency, but also in patients with high preop-eGFR.

## Methods

### Data source

We downloaded the raw data from the DATADRYAD database (www.datadryad.org). As Diana Xin Hui Chan, et al. [17] have uploaded the original data and authorized the ownership to the website, we can perform secondary data analysis on this data to verify different scientific assumptions. (Dryad data package: Chan et al. [17], Data from: Development of the Combined Assessment of Risk Encountered in Surgery (CARES) surgical risk calculator for prediction of post-surgical mortality and need for intensive care unit admission risk—a single-center retrospective study, Dryad, Dataset, https://doi.org/10.5061/dryad.v142481).

### Study population

It is important to note that Chan et al. [17] completed data collection. They conducted a single-center retrospective study at Singapore General Hospital, a 1700-bed tertiary academic hospital [17]. These clinical data of all surgical patients from January 1, 2012 to October 31, 2016 were obtained from the clinical information system (Sunrise Clinical Manager, Allscripts, Illinois, USA) and stored in SingHealth-IHiS Electronic Health Information System (eHINTS), a data storage and analysis system. Based on exclusion criteria, the study recruited a total of 79,609 surgical cases [17]. Exclusion standards: (1) patients with no surgery performed; (2) patients undergoing cardiac surgery, neurosurgery, transplantation and burn surgery; (3) pediatric cases; (4) cases under local anesthesia; (5) cases under cadaveric harvesting; (6) cases with missing important variables. As the personal information of patients was anonymous, no informed consent was required. Details of ethical license can be found in the data source article [17].

### Measurement of preop-eGFR, 30-day mortality and other covariates

Variables of the database file included: demographic information; preoperative comorbidities; preoperative laboratory; perioperative blood transfusion data; anesthesia type;priority of surgery; surgical risk classification (based on the 2014 ESC/ESA guidelines on non-cardiac surgery [18, 19]); and postoperative 30-day prognosis. Preoperative comorbidities consisted of anemia, CKD, and medical history [cerebrovascular accidents (CVA), ischemic heart disease (IHD), congestive heart failure (CHF), type 1 diabetes]. Those medical histories mentioned above were also associated with Revised Cardiac Risk Index (RCRI) score [20]. The latest results of preoperative laboratory test were mainly about eGFR, red blood cell distribution width (RDW) (Levels above 15.7% were defined as high RDW, as the normal reference range was 10.9% to 15.7% [21]), hemoglobin. We listed in detail the covariates used in this study. In short, inclusionary criteria were the following: (1) demographic data; (2) variables that can affect preop-eGFR or 30-day mortality reported by previous literature [17]; (4) based on our clinical experiences. The most important outcome was postoperative 30-day mortality, including deaths from the date of surgery to 1 month later [3]. As this is a retrospective study, reducing the possibility of selection bias and observation bias.

### Statistical analysis

In data analysis, we represented continuous variables as median (quartile) (skewed distribution) or mean ± standard deviation (normal distribution), and categorical variables as a percentage or frequency. In the process of multivariate regression analysis, there are some confounders with partial missing data. If it is a categorical variable, the missing data would be treated directly as a new independent group; if it is a continues variable, the missing data would be replaced with an average or median value. We use Kruskal–Wallis H test (skewed distribution), one-way ANOVA (normal distribution) or χ^2^ (categorical variables) to calculate differences between different preop-eGFR group.

Our study supposed to figure out the specific relationship between preop-eGFR and postoperative 30-day mortality (linear or non-linear), and then find out variables interfering with or modifying the relationship between them. After excluding the effects of these potential modifiers and confounders, the independent effect of preop-eGFR on postoperative 30-day mortality can be determined.

Based on analytical principles mentioned above, univariate and multiple linear regression models were used to assess relationships with preop-eGFR and postoperative 30-day mortality. Three models (an unadjusted model, a demographically adjusted model, and a fully-adjusted model) were constructed according to the STROBE statement [22]. As for the fully-adjusted model, those adjusted variables, reported in previous studies, are related covariates that may affect preop-eGFR and/or 30-day mortality [17, 21]. Moreover, subgroup analyses were performed by stratified linear regression models. Subgroup interaction test was performed to verity the effect modification by subgroup and then a likelihood ratio test was conducted.

The following sensitivity analysis was performed in our research to ensure that the results of the data analysis were reliable. Firstly, the continuous variable preop-eGFR was converted into a categorical one by quartile to observe the possibility of nonlinearity. Secondly, if a nonlinear relationship exited between preop-eGFR and postoperative 30-day mortality, it would be processed using a generalized additive model. Thirdly, we measured the threshold effect of preop-eGFR on the 30-day mortality rate via a two-segment linear regression model based on a smoothing graph, determined the saturation of preop-eGFR by a recursive algorithm, and then detected the inflection point to obtain the maximum model likelihood. Based on the P-value of the log-likelihood ratio test, the best-fit model can be determined.

All the analyses were performed with the statistical software packages R (The R Foundation, http://www.r-project.org) and EmpowerStats (X&Y Solutions, Inc., Boston, MA, http://www.empowerstats.com). P-value < 0.05 was considered statistically significant.

## Results

### The selection of participants

The original data (N = 90,785) for this study was recruited according to exclusion standards by Chan et al. [17]. The missing data on preop-eGFR and 30-day mortality was about 10,830 cases. And after excluding patients with preop-eGFR outliers [23] (N = 346), 79,609 cases were included to our study (shown in Schedule 1).

### Baseline characteristics of participants

Baseline characteristics of the 79,609 participants based on the quartiles of preop-eGFR are shown in Table 1. Overall, the average age of all patients participating in the study was 54.01 ± 16.86 years old, of which approximately 48.47% were male. The participants’ preop-eGFR distribution was mean 84.45 ± 38.56 (min 2.004, max 206.225). The 30-day postoperative death in the study population was 506 cases (0.64%). The numbers of participants with missing data for each variable are shown in Table 1. Compared with participants with lower preop-eGFR (Q1–Q3), highest preop-eGFR was significantly positively correlated with females, younger age transfusion, emergency surgery, general anesthesia, comorbidities, but lower rate of Chinese (All P-values < 0.001). There were also statistically differences in anemia and RDW with different preop-eGFR. When the intermediate level of preop-eGFR (Q2–3) was reached, the patient was more likely to have none anemia and RDW ≤ 15.7% (P-value < 0.001).

**Table 1:** Baseline characteristics of participants

Preop-eGFR (quartile)	Q1 (31.61 ± 18.07)	Q2 (78.69 ± 6.52)	Q3 (96.68 ± 4.67)	Q4 (130.78 ± 20.86)	P-value
N (cases)	19,901	19,882	19,915	19,911	
Age Year	57.72 ± 17.41	59.96 ± 14.04	52.26 ± 15.56	46.08 ± 16.75	< 0.001
Sex N (%)					< 0.001
Male	9535 (47.91%)	11,493 (57.81%)	10,334 (51.89%)	7199 (36.16%)	
Female	10,366 (52.09%)	8389 (42.19%)	9581 (48.11%)	12,712 (63.84%)	
Race N (%)					< 0.001
Chinese	14,592 (73.33%)	14,943 (75.16%)	14,403 (72.32%)	14,005 (70.34%)	
Indian	1540 (7.74%)	1602 (8.06%)	1809 (9.08%)	1875 (9.42%)	
Malay	2166 (10.88%)	1679 (8.44%)	1748 (8.78%)	1970 (9.89%)	
Others	1601 (8.05%)	1658 (8.34%)	1955 (9.82%)	2060 (10.35%)	
Postop-transfusion within 30 days					< 0.001
0 units	19,505 (98.01%)	19,752 (99.35%)	19,815 (99.50%)	19,647 (98.67%)	
1 unit	244 (1.23%)	81 (0.41%)	74 (0.37%)	177 (0.89%)	
2 or more units	152 (0.76%)	49 (0.25%)	26 (0.13%)	87 (0.44%)	
Intraop-transfusion					< 0.001
0 units	18,163 (91.27%)	18,985 (95.49%)	19,184 (96.33%)	18,629 (93.56%)	
1 unit	1738 (8.73%)	897 (4.51%)	731 (3.67%)	1282 (6.44%)	
Preop-transfusion within 30 days					< 0.001
0 units	19,142 (96.19%)	19,551 (98.34%)	19,683 (98.84%)	19,484 (97.86%)	
1 unit	374 (1.88%)	186 (0.94%)	140 (0.70%)	241 (1.21%)	
2 or more units	385 (1.93%)	145 (0.73%)	92 (0.46%)	186 (0.93%)	
Anemia category N (%)					< 0.001
None	11,532 (57.95%)	15,561 (78.27%)	16,381 (82.25%)	14,309 (71.86%)	
Mild	3887 (19.53%)	2738 (13.77%)	2197 (11.03%)	2939 (14.76%)	
Moderate and severe	4438 (22.30%)	1540 (7.75%)	1287 (6.46%)	2611 (13.11%)	
NA	44 (0.22%)	43 (0.22%)	50 (0.25%)	52 (0.26%)	
Priority of surgery N (%)					< 0.001
Elective	14,929 (75.02%)	16,786 (84.43%)	16,713 (83.92%)	15,155 (76.11%)	
Emergency	4972 (24.98%)	3096 (15.57%)	3202 (16.08%)	4756 (23.89%)	
Anesthesia type N (%)					< 0.001
GA	15,803 (79.41%)	16,384 (82.41%)	17,453 (87.64%)	17,715 (88.97%)	
RA	4098 (20.59%)	3498 (17.59%)	2462 (12.36%)	2196 (11.03%)	
Surgical risk					< 0.001
Low	9925 (49.87%)	10,165 (51.13%)	10,705 (53.75%)	10,079 (50.62%)	
Moderate	8962 (45.03%)	8892 (44.72%)	8599 (43.18%)	9047 (45.44%)	
High	1014 (5.10%)	825 (4.15%)	611 (3.07%)	785 (3.94%)	
RDW N (%)					< 0.001
RDW ≤ 15.7%	17,300 (87.38%)	18,448 (93.27%)	18,377 (92.73%)	17,368 (87.66%)	
RDW > 15.7%	2498 (12.62%)	1332 (6.73%)	1440 (7.27%)	2444 (12.34%)	
CVA category					< 0.001
No	13,231 (66.48%)	13,348 (67.14%)	13,771 (69.15%)	13,558 (68.09%)	
Yes	640 (3.22%)	481 (2.42%)	211 (1.06%)	168 (0.84%)	
NA	6030 (30.30%)	6053 (30.44%)	5933 (29.79%)	6185 (31.06%)	
IHD category					< 0.001
No	11,914 (59.87%)	12,581 (63.28%)	13,314 (66.85%)	13,264 (66.62%)	
Yes	1890 (9.50%)	1205 (6.06%)	616 (3.09%)	406 (2.04%)	
NA	6097 (30.64%)	6096 (30.66%)	5985 (30.05%)	6241 (31.34%)	
CHF category					< 0.001
No	13,934 (70.02%)	14,269 (71.77%)	14,399 (72.30%)	14,022 (70.42%)	
Yes	489 (2.46%)	147 (0.74%)	64 (0.32%)	70 (0.35%)	
NA	5478 (27.53%)	5466 (27.49%)	5452 (27.38%)	5819 (29.23%)	
Dminsulin category					< 0.001
No	13,235 (66.50%)	13,932 (70.07%)	14,134 (70.97%)	13,670 (68.66%)	
Yes	1074 (5.40%)	340 (1.71%)	195 (0.98%)	301 (1.51%)	
NA	5592 (28.10%)	5610 (28.22%)	5586 (28.05%)	5940 (29.83%)	
RCRI score					< 0.001
I	8549 (42.96%)	10,605 (53.34%)	11,564 (58.07%)	10,864 (54.56%)	
II	3571 (17.94%)	2935 (14.76%)	2351 (11.81%)	2727 (13.70%)	
III	1376 (6.91%)	520 (2.62%)	237 (1.19%)	238 (1.20%)	
IV	685 (3.44%)	100 (0.50%)	43 (0.22%)	41 (0.21%)	
NA	5720 (28.74%)	5722 (28.78%)	5720 (28.72%)	6041 (30.34%)	
30-day mortality N (%)					< 0.001
No	19,587 (98.42%)	19,800 (99.59%)	19,880 (99.82%)	19,836 (99.62%)	
Yes	314 (1.58%)	82 (0.41%)	35 (0.18%)	75 (0.38%)	

*GA* general anesthesia, *RA* regional anesthesia, *preop-eGFR* preoperative estimated glomerular filtration rates, *RDW* red cell distribution, *NA* not available, *CVA* cerebrovascular accidents, *IHD* ischemic heart disease, *CHF* congestive heart failure, *DM* diabetes mellitus requiring insulin therapy, *Preop* preoperative, *Intraop* intraoperative, *Postop* postoperative, *RCRI* Revised Cardiac Risk Index, *ASA* American Society of Anesthesiologists

### Univariate and multivariate analysis

Univariate analysis results were seen in Table 2. These results indicates that age, transfusion rate frequency, anemia, priority of surgery, surgical risk, RDW and comorbidities (CVA, IHD, CHF, DM on insulin) was correlated with higher 30-day mortality (All P-values < 0.05). We also find that there was no significant difference of 30-day mortality with different race (P-value > 0.05), whereas preop-eGFR and female were related to 30-day mortality with a statistically significant negative correlation (All P-values < 0.05).

**Table 2:** Effects of risk factors of 30-day mortality

Preop-eGFR (quartile)	Statistics	OR	95% CI	P value
Age Mean (STD), Year	53.96 ± 16.88	1.07	(1.06, 1.08)	< 0.0001
Sex N (%)				
Male	38,666 (48.36%)	1.0		
Female	41,289 (51.64%)	0.69	(0.58, 0.82)	< 0.0001
Race N (%)				
Chinese	58,170 (72.76%)	1.0		
Indian	6860 (8.58%)	0.91	(0.66, 1.25)	0.5640
Malay	7616 (9.53%)	1.23	(0.94, 1.61)	0.1250
Others	7305 (9.14%)	0.63	(0.44, 0.91)	0.0134
Postop-transfusion within 30 days				
0 units	79,026 (98.84%)	1.0		
1 unit	595 (0.74%)	10.63	(7.43, 15.21)	< 0.0001
2 or more units	334 (0.42%)	27.31	(19.68, 37.90)	< 0.0001
Intraop-transfusion				
0 units	75,228 (94.09%)	1.0		
1 unit	4727 (5.91%)	10.72	(8.98, 12.80)	< 0.0001
Preop-transfusion within 30 days				
0 units	78,160 (97.75%)	1.0		
1 unit	963 (1.20%)	12.78	(9.60, 17.00)	< 0.0001
2 or more units	832 (1.04%)	23.71	(18.59, 30.26)	< 0.0001
Anemia category N (%)				
None	57,906 (72.42%)	1.0		
Mild	11,816 (14.78%)	4.48	(3.40, 5.89)	< 0.0001
Moderate and severe	10,044 (12.56%)	17.55	(14.10, 21.86)	< 0.0001
NA	189 (0.24%)	5.72	(1.40, 23.35)	0.0150
Priority of surgery N (%)				
Elective	63,770 (79.76%)	1.0		
Emergency	16,185 (20.24%)	7.87	(6.57, 9.44)	< 0.0001
Anesthesia type N (%)				
General anaesthesia	67,640 (84.60%)	1.0		
Regional anaesthesia	12,315 (15.40%)	1.18	(0.94, 1.48)	0.1525
Surgical risk				
Low	41,001 (51.28%)	1.0		
Moderate	35,692 (44.64%)	2.31	(1.89, 2.82)	< 0.0001
High	3262 (4.08%)	7.53	(5.75, 9.84)	< 0.0001
RDW N (%)				
RDW ≤ 15.7%	71,714 (89.69%)	1.0		
RDW > 15.7%	7839 (9.80%)	7.00	(5.88, 8.34)	< 0.0001
NA	402 (0.50%)	1.79	(0.57, 5.60)	0.3175
CVA category				
No	54,149 (67.72%)	1.0		
Yes	1507 (1.88%)	5.60	(4.03, 7.77)	< 0.0001
NA	24,299 (30.39%)	1.69	(1.41, 2.03)	< 0.0001
IHD category				
No	51,310 (64.17%)	1.0		
Yes	4126 (5.16%)	8.32	(6.63, 10.44)	< 0.0001
NA	24,519 (30.67%)	2.30	(1.89, 2.80)	< 0.0001
CHF category				
No	56,876 (71.14%)	1.0		
Yes	776 (0.97%)	9.15	(6.40, 13.09)	< 0.0001
NA	22,303 (27.89%)	1.75	(1.46, 2.10)	< 0.0001
Dminsulin category				
No	55,206 (69.05%)	1.0		
Yes	1929 (2.41%)	3.98	(2.84, 5.57)	< 0.0001
NA	22,820 (28.54%)	1.73	(1.44, 2.07)	< 0.0001
Preop-eGFR	84.45 ± 38.56	0.98	(0.98, 0.98)	< 0.0001

The results of univariate and multivariate linear regression models are shown in Table 3. The unadjusted model showed a 30-day mortality reduction of 1.8% for each additional preop-eGFR unit (95% CI 0.979, 0.984). It also illustrates different kinds of adjusted model including minimally-adjusted, fully-adjusted model. Compared to the unadjusted model (OR = 0.982, 95% CI 0.979, 0.984), the preop-eGFR in the minimal adjustment model (only adjusted for age, sex, and race) still negatively related to the 30-day mortality (OR = 0.983, 95% CI 0.980, 0.986). In the fully-adjusted model, preop-eGFR significantly functioned as the same but with a little increase in the effect size (OR = 0.992, 95% CI 0.990, 0.995).

**Table 3:** The results of univariate and multivariate analyses

Exposure	Model 1	Model 2	Model 3
Preop-eGFR	0.982 (0.979, 0.984) < 0.00001	0.983 (0.980, 0.986) < 0.00001	0.992 (0.990, 0.995) < 0.00001
Preop-eGFR (quartile)			
Q1 (31.61 ± 18.07)	Ref	Ref	Ref
Q2 (78.69 ± 6.52)	0.258 (0.202, 0.330) < 0.00001	0.256 (0.201, 0.328) < 0.00001	0.520 (0.401, 0.675) < 0.00001
Q3 (96.68 ± 4.67)	0.110 (0.077, 0.156) < 0.00001	0.169 (0.119, 0.241) < 0.00001	0.356 (0.247, 0.514) < 0.00001
Q4 (130.78 ± 20.86)	0.236 (0.183, 0.304) < 0.00001	0.516 (0.396, 0.671) < 0.00001	0.616 (0.468, 0.812) 0.00058
P for trend	< 0.00001	< 0.00001	< 0.00001

Results is showed in OR (95% CI) P-value (*OR* odd rates, *CI* confidence interval)

Model 1 (Non-adjusted model): not adjust any covariate

Model 2 (Minimally-adjusted model): only adjusted age, sex and race

Model 3 (fully-adjusted model): adjusted age, sex, race, anesthesia type, Revised Cardiac Risk Index (RCRI) score, Transfusion Intra and postop Category, preop-transfusion within 30 days, anesthesia type, priority of surgery, surgical risk, and RDW

In the sensitivity analysis, we converted preop-eGFR to a categorical variable classified by quartile to observe P of trend (Table 3). Compared to the reference group (Q1), the effect size of preop-eGFR on 30-day mortality in group Q3 (preop-eGFR: 96.68 ± 4.67) was the smallest among different adjusted model. For instance, in the fully-adjusted model, the effect size of group Q2, Q3 and Q4 were 0.520, 0.356, 0.616 respectively, compared with group (Q1). This kind of non-equidistant changes in effect size indicated a non-linear relationship between preop-eGFR with 30-day mortality.

### The analyses of non-linear relationship

**Fig. 1:**
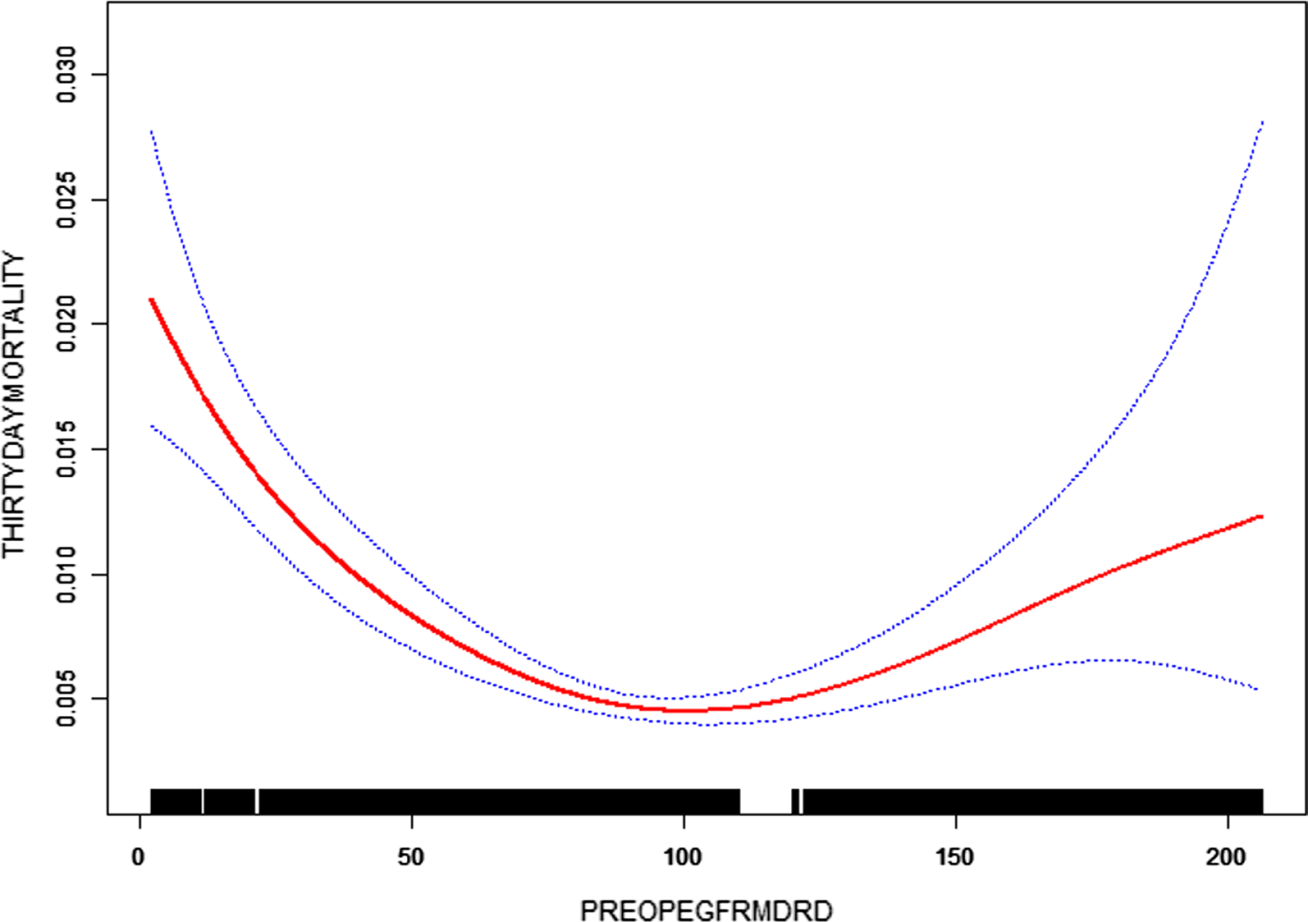
Relationship of e-GFR and 30-day mortality (dotted line: 95% confidence interval; solid line: odds ratio)

Figure 1 shows the U-shaped non-linear correlation between preop-eGFR and postoperative 30-day mortality. This non-linear relationship was verified by smooth curve of the generalized additive model. And the P-value of the log likelihood ratio test is less than 0.05 in Table 4, which further indicates the two-part linear regression model should be used to fit the relationship, rather than the linear regression model (through linearly fitting). The inflection point was calculated to be 98.688 by a two-part linear regression model and a recursive algorithm. On the left side of the inflection point, preop-eGFR is one of the independently protective factors of 30-day mortality (OR = 0.984, 95% CI 0.981 to 0.987, P < 0.0001). While on the right side, it acts as a risk factor (OR = 1.013, 95% CI 1.007 to 1.019, P < 0.0001).

**Table 4:** The results of two-piecewise linear regression

	30-day mortality (OR, 95% CI, P-value)
Inflection point of preop-eGFR	98.688
≤ 98.688	0.984 (0.981, 0.987) < 0.0001
> 98.688	1.013 (1.007, 1.019) < 0.0001
P-value for log likelihood ratio test	< 0.001

Adjusted age, sex, race, the frequency of transfusion (including preoperative, intraoperative, postoperative), priority of surgery, anesthesia type, surgical risk, red cell distribution width, anemia category, and comorbidities such as DM on insulin, CVA, CHF, IHD

### The results of subgroup analyses and interaction analysis

As is shown in Table 5, the interaction test was significant for patients comorbidities, as DM, CHF, IHD or anemia (P = 0.0397, 0.0357, 0.0168, < 0.0001 for interaction, respectively), while the interaction of other covariates was not statistically significant (interaction P value is greater than 0.05). Under the influence of preoperative comorbidities, such as CHF, DM and IHD, the effect of preop-eGFR on the 30-day postoperative mortality gradually increased. For patients with CHF, an increase of the preop-eGFR unit related to a 1.2% reduction in 30-day mortality (0.988 (0.984, 0.992)). Without CHF, there’s decreased by 0.5% for each additional unit of preop-eGFR (0.995 (0.992, 0.998)). The same trend is also observed in in patients with DM (1.3% reduction of 30-day mortality with DM vs. 0.5% reduction without DM) and IHD (0.9% with IHD vs. 0.4% without IHD). However, the opposite trend was seen in people with anemia. For patients without anemia, an increase unit of preop-eGFR would cost a 2.3% reduction in 30-day mortality (0.977, 95% CI 0.971, 0.984). For patients with anemia, the 30-day mortality was decreased by 0.5% with each additional unit of preop-eGFR.

**Table 5:** Results of interaction analysis and subgroup analysis

Characteristic	OR (95% CI)2	P for interaction2
Age (Mean ± STD)		0.1128
Q1 (26.22 ± 6.97)	1.002 (0.984, 1.019)	
Q2 (41.20 ± 3.78)	0.996 (0.988, 1.003)	
Q3 (53.26 ± 3.16)	0.999 (0.993, 1.005)	
Q4 (62.98 ± 2.55)	0.991 (0.986, 0.996)	
Q5 (75.07 ± 5.69)	0.990 (0.987, 0.994)	
Sex N (%)		0.2265
Male	0.994 (0.990, 0.997)	
Female	0.991 (0.987, 0.994)	
Race N (%)		0.5579
Chinese	0.991 (0.989, 0.994)	
Indian	0.991 (0.982, 1.000)	
Malay	0.996 (0.988, 1.003)	
Others	0.997 (0.987, 1.008)	
Postop-transfusion within 30 days		0.4309
0 units	0.992 (0.990, 0.995)	
1 unit	0.997 (0.987, 1.007)	
2 or more units	0.988 (0.978, 0.997)	
Intraop-transfusion		0.1152
0 units	0.991 (0.988, 0.994)	
1 unit	0.995 (0.991, 0.998)	
Preop-transfusion within 30 days		0.1362
0 units	0.991 (0.988, 0.994)	
1 unit	0.992 (0.984, 1.000)	
2 or more units	0.997 (0.992, 1.003)	
Anemia category N (%)		< 0.0001
None	0.977 (0.971, 0.984)	
Mild	0.995 (0.989, 1.001)	
Moderate and severe	0.995 (0.992, 0.998)	
Priority of surgery N (%)		0.0742
Elective	0.995 (0.991, 0.999)	
Emergency	0.991 (0.988, 0.994)	
Anesthesia type N (%)		0.2401
General anaesthesia	0.993 (0.990, 0.996)	
Regional anaesthesia	0.989 (0.983, 0.995)	
Surgical risk		0.0755
Low	0.988 (0.983, 0.993)	
Moderate	0.995 (0.991, 0.998)	
High	0.992 (0.986, 0.998)	
RDW N (%)		0.8496
RDW ≤ 15.7%	0.992 (0.989, 0.996)	
RDW > 15.7%	0.992 (0.989, 0.996)	
CVA category		0.0884
No	0.995 (0.992, 0.998)	
Yes	0.989 (0.979, 0.999)	
IHD category		0.0283
No	0.996 (0.992, 1.000)	
Yes	0.991 (0.985, 0.997)	
CHF category		0.0357
No	0.995 (0.992, 0.998)	
Yes	0.988 (0.984, 0.992)	
Dminsulin category		0.0397
No	0.995 (0.992, 0.998)	
Yes	0.987 (0.976, 0.998)	

Above model is not adjusted for the stratification variable

In each case, the model adjusted for age, sex, race, anesthesia type, Revised Cardiac Risk Index (RCRI) score, Transfusion Intra and postop Category, preop-transfusion within 30 days, anesthesia type, priority of surgery, surgical risk, and RDW

## Discussion

In this study, we confirmed an independent nonlinear relationship between preop-eGFR and postoperative 30-day mortality. A stable U-shaped trend can be seen in this relationship. When preop-eGFR ≤ 98.688 mL/min/1.73 m^2^, the 30-day mortality would be decreased by 1.6% for each additional unit of preop-eGFR. While preop-eGFR > 98.688 mL/min/1.73 m^2^, there would be a 1.3% increase of 30-day mortality rate for each additional unit of preop-eGFR. CHF, DM, IHD and anemia complications (CHF, DM, IHD acting as a promoting factor, while anemia as an inhibitory factor) interfere with the effect of preop-eGFR on postoperative 30-day mortality.

Previous studies have also supported the hypothesis that preop-eGFR is a powerful and independent predictor of 30-day morbidity risk after surgery [4, 10, 13, 15, 16, 24, 25]. Even in some literature reports, preop-eGFR is the strongest predictors of posttransplant survival [24]. Preop-eGFRs is a important indicator of many adverse surgical outcomes [16], as acute kidney injury, significantly related to higher mortality. At the same time, DM, IHD, CVF, and blood transfusion also are risk factors for poor postoperative prognosis [6]. The current research population is mainly concentrated in transplant [24], cardiac [10, 13] and neuro [14] surgery. There is still a lack of research on other surgery. There are two articles that define the study population as non-cardiac surgery patients. Cywinski, et al. evaluated 92,888 patients undergoing non-cardiac surgery, and confirmed preop-eGFR is a scientifically feasible predictor of postoperative 30-day mortality [4]. Prowle et al. reported that significantly increases the risk of death after non-cardiac surgery, according to the data of 36,779 cases [2].

Previous studies mostly focused on the patients of renal insufficiency to verify the important regulatory role of preop-eGFR [12, 13, 15]. High preop-eGFR levels have also been connected with greater mortality among nonsurgical patients indicating a potential U-shaped association of preop-eGFR with poor prognosis [26–28]. A recent study revealed the association of the specific trend between preop-eGFR and 30-day mortality in patients undergoing surgery for gastrointestinal malignancies, without clarifying the inflection point [16]. In addition, the current research population is mainly Europeans and Americans, and rarely Asians.

To our best knowledge, it is the first time that the specific U-shaped relationship between preop-eGFR and postoperative 30-day mortality has been clearly identified in Asian patients undergoing non-cardiac and non-neuro surgery, ranging from minor day cases to major surgeries.

Strengths of our study are mentioned as follows: firstly, the generalized additive model was used to evaluate non-linear relations, instead of using the generalized linear model to illustrate the linear relationship only. Secondly, as an observational study, there were some unavoidable potential confounders included in this study. In order to minimize residual confounding, strict statistical adjustment was performed. What’s more, effect modifier factor analysis ameliorates the use of data. Sensitivity analysis was performed of these data to ensure reliability.

The findings of this study should be helpful for reducing the risk of postoperative death. The preop-eGFR at which the rate of the perioperative Mortality was lowest was 98.688. It suggests that regulation of preop-eGFR can effectively reduce perioperative mortality, especially with CHF, DM, IHD comorbidities. While has comorbid anemia, it also becomes quite important to control anemia for reducing mortality.

This study has several acknowledged limitations. First, as for our study is a secondary analysis based on the published data, we cannot exclude some residual and/or unmeasured confounders (such as socioeconomic factors and inflammatory markers), that may bias the estimated relationship. Secondly, the study population, which only included Asian patients, can be further expanded to conduct multi-center research to increase the reliability of the data. Our choice of outcomes and variables is also limited. We could not investigate the relationship between preop-eGFR with long-term outcomes. What’s more, when it comes to high preop-eGFR, the results would be much more accurate formula based on cystatin C, instead of basing on creatinine. However, cystatin C haven’t be widely used in clinical practice right now [29].

## Conclusion

In patients undergoing non-cardiac and non-neurological surgery, the level of preop-eGFR is associated with operative adverse events in a U-shape trend. The preop-eGFR with the lowest perioperative mortality was 98.688. CHF, DM, IHD and anemia comorbidities (CHF, DM, IHD as a promoting factor, anemia as an inhibitor) interfere with the effect of preop-eGFR on postoperative 30-day mortality.

## Data Availability

The raw data can be downloaded from the website (www.Datadryad.org) of ‘Datadryad’ database.

## Abbreviations

Preop-eGFR: Preoperative estimated glomerular filtration rate
CHF: Congestive heart failure
IHD: Ischemic heart disease
DM: Diabetes mellitus
OR: Odds ratio
CI: Confidence interval
POMR: Perioperative mortality
CKD: Chronic kidney disease
CARES: Combined Assessment of Risk Encountered in Surgery
RCRI score: Revised Cardiac Risk Index Score
RDW: Red cell distribution width
eHINTS: SingHealth-IHiS Electronic Health Information System
RP: Renal group
RBC: Red blood cell
FBC: Full blood count
MDRD: Modification of diet in renal disease
WHO: World Health Organization
KDIGO: Kidney disease: improving global outcomes
ANOVA: Analysis of variance
STROBE: Strengthening the Reporting of Observational Studies in Epidemiology
Q1–4: Quartile 1–4
GAM: Generalized additive model

## References

[1] Weiser TG, Haynes AB, Molina G, Lipsitz SR, Esquivel MM, Uribe-Leitz T, Fu R, Azad T, Chao TE, Berry WR, Gawande AA (2015). Estimate of the global volume of surgery in 2012: an assessment supporting improved health outcomes. Lancet.

[2] Prowle JR, Kam EP, Ahmad T, Smith NC, Protopapa K. Preoperative renal dysfunction and mortality after non-cardiac surgery. Br J Surg. 2016;103(10):1316–25. 10.1002/bjs.10186 27346181

[3] Watters DA, Hollands MJ, Gruen RL, Maoate K, Perndt H, McDougall RJ, Morriss WW, Tangi V, Casey KM, McQueen KA (2015). Perioperative mortality rate (POMR): a global indicator of access to safe surgery and anaesthesia. World J Surg.

[4] Cywinski JB, Mascha EJ, Kurz A, Sessler DI (2015). Estimated glomerular filtration rate better predicts 30-day mortality after non-cardiac surgery than serum creatinine: a retrospective analysis of 92,888 patients. Can J Anesth.

[5] Warnock DG (2016). Estimated glomerular filtration rate: fit for what purpose?. Nephron.

[6] Thongprayoon C, Cheungpasitporn W, Mao MA, Srivali N, Kittanamongkolchai W, Harrison AM, Greason KL, Kashani KB (2017). Persistent acute kidney injury following transcatheter aortic valve replacement. J Cardiac Surg.

[7] Yamamoto M, Hayashida K, Mouillet G, Hovasse T, Chevalier B, Oguri A, Watanabe Y, Dubois-Randé J, Morice M, Lefèvre T, Teiger E (2013). Prognostic value of chronic kidney disease after transcatheter aortic valve implantation. J Am Coll Cardiol.

[8] Jang MS, Nam JS, Jo JY, Kang CH, Ryu SA, Lee EH, Choi IC (2019). The relationship of preoperative estimated glomerular filtration rate and outcomes after cardiovascular surgery in patients with normal serum creatinine: a retrospective cohort study. BMC Anesthesiol.

[9] Dardashti A, Nozohoor S, Algotsson L, Ederoth P, Bjursten H (2016). The predictive value of s-cystatin C for mortality after coronary artery bypass surgery. J Thorac Cardiovasc Surg.

[10] Bissacco D, Catanese V, Fossati A, Salvati S, Zanella G, Carmo M, Settembrini P (2018). Role of preoperative serum creatinine and estimated glomerular filtration rate values in asymptomatic patients undergoing carotid endarterectomy. J Cardiovasc Surg (Torino).

[11] Mooney JF, Croal BL, Cassidy S, Lee VW, Chow CK, Cuthbertson BH, Hillis GS (2019). Relative value of cystatin C and creatinine-based estimates of glomerular filtration rate in predicting long-term mortality after cardiac surgery: a cohort study. BMJ Open.

[12] Wang W, Wang Y, Xu R, Chai J, Zhou W, Chen H, Wang K, Kong X (2018). Outcomes following coronary artery bypass graft surgery in patients with mild preoperative renal insufficiency. Braz J Cardiovasc Surg.

[13] Bernardi MH, Schmidlin D, Schiferer A, Ristl R, Neugebauer T, Hiesmayr M, Druml W, Lassnigg A (2015). Impact of preoperative serum creatinine on short- and long-term mortality after cardiac surgery: a cohort study. Br J Anaesth.

[14] Kim K, Bang J, Kim S, Kim S, Kim JU, Song J (2018). Association of preoperative hypoalbuminemia with postoperative acute kidney injury in patients undergoing brain tumor surgery: a retrospective study. J Neurosurg.

[15] Martin CT, Pugely AJ, Gao Y, Mendoza-Lattes SA, Weinstein SL (2015). The impact of renal impairment on short-term morbidity risk following lumbar spine surgeries. Spine.

[16] Ui T, Obi Y, Shimomura S, Lefor AK, Alizadeh RF, Said H, Nguyen NT , Stamos MJ, Kalantar-Zadeh K, Sata N, Ichii H. High and low estimated glomerular filtration rates are associated with adverse outcomes in patients undergoing surgery for gastrointestinal malignancies. Nephrol Dial Transplant. 2019;34(5):810–818. 10.1093/ndt/gfy108 29718365

[17] Chan DXH, Sim YE, Chan YH, Poopalalingam R, Abdullah HR. Development of the combined assessment of risk encountered in surgery (CARES) surgical risk calculator for prediction of postsurgical mortality and need for intensive care unit admission risk: a single-center retrospective study. BMJ Open. 2018;8:e019427. 10.1136/bmjopen-2017-019427 PMC5875658 29574442

[18] Glance LG, Lustik SJ, Hannan EL, Osler TM, Mukamel DB, Qian F, Dick AW (2012). The surgical mortality probability model. Ann Surg.

[19] Kristensen SD, Knuuti J, Saraste A, Anker S, Bøtker HE, De Hert S, Ford I, Juanatey JRG, Gorenek B, Heyndrickx GR, Hoeft A, Huber K, Iung B, Kjeldsen KP, Longrois D, Luescher TF, Pierard L, Pocock S, Price S, Roffi M, Sirnes PA, Uva MS, Voudris V, Funck-Brentano C (2014). ESC/ESA guidelines on non-cardiac surgery. Eur J Anaesth.

[20] Derivation and prospective validation of a simple index for prediction of cardiac risk of. 10.1161/01.cir.100.10.1043 10477528

[21] Sim YE, Wee HE, Ang AL, Ranjakunalan N, Ong BC, Abdullah HR (2017). Prevalence of preoperative anemia, abnormal mean corpuscular volume and red cell distribution width among surgical patients in Singapore, and their influence on one year mortality. PLoS ONE.

[22] Fitchett EJA, Seale AC, Vergnano S, Sharland M, Heath PT, Saha SK, Agarwal R, Ayede AI, Bhutta ZA, Black R, Bojang K, Campbell H, Cousens S, Darmstadt GL, Madhi SA, Meulen AS, Modi N, Patterson J, Qazi S, Schrag SJ, Stoll BJ, Wall SN, Wammanda RD, Lawn JE (2016). Strengthening the reporting of observational studies in epidemiology for newborn infection (STROBE-NI): an extension of the STROBE statement for neonatal infection research. Lancet Infect Dis.

[23] Zhang N, Hu X, Zhang Q, Bai P, Cai M, Zeng TS, Zhang J-Y, Tian S-H, Min J, Huang H-T, Zheng J, Peng M-M, Li M-J, Chen L-L. Non‐high‐density lipoprotein cholesterol:High‐density lipoprotein cholesterol ratio is an independent risk factor for diabetes mellitus: Results from a population‐based cohort study. J Diabetes. 2018;10(9):708–714. 10.1111/1753-0407.12650 29437292

[24] Sargut TA, Pergantis P, Knosalla C, Knierim J, Hummel M, Falk V, Schoenrath F (2018). Adjusting preoperative risk models of post heart transplant survival to a European cohort in the age of a new cardiac allocation score in Europe. Heart Surg Forum.

[25] Padhy S (2017). Prediction of major adverse cardiovascular and cerebrovascular events (MACCE) after thoracic surgery-the role of estimated GFR. J Clin Diagn Res.

[26] Association of estimated glomerular filtration rate and albuminuria with all-cause and cardiovascular mortality in general population cohorts: a collaborative meta-analysis. Lancet. 2010;375: 2073–2081. 10.1016/S0140-6736(10)60674-5 PMC3993088 20483451

[27] Tonelli M, Klarenbach SW, Lloyd AM, James MT, Bello AK, Manns BJ, Hemmelgarn BR (2011). Higher estimated glomerular filtration rates may be associated with increased risk of adverse outcomes, especially with concomitant proteinuria. Kidney Int.

[28] Matsushita K, Mahmoodi BK, Woodward M, Emberson JR, Jafar TH, Jee SH, Polkinghorne KR, Shankar A, Smith DH, Tonelli M, Warnock DG, Wen C, Coresh J, Gansevoort RT, Hemmelgarn BR, Levey AS, F.T. Chronic Kidney Disease Prognosis Consortium (2012). Comparison of risk prediction using the CKD-EPI equation and the MDRD study equation for estimated glomerular filtration rate. JAMA.

[29] Levey AS, Becker C, Inker LA (2015). Glomerular filtration rate and albuminuria for detection and staging of acute and chronic kidney disease in adults. JAMA.

[30] Levey AS, Eckardt K, Tsukamoto Y, Levin A, Coresh J, Rossert J, Zeeuw DDE, Hostetter TH, Lameire N, Eknoyan G (2005). Definition and classification of chronic kidney disease: A position statement from Kidney Disease: Improving Global Outcomes (KDIGO). Kidney Int.

[31] WHO_TRS_405.

